# Editorial: What Do We Know About Other Specified Feeding or Eating Disorders, Unspecified Feeding and Eating Disorder and the Other EXIAs (e.g., Orthorexia, Bigorexia, Drunkorexia, Pregorexia etc.)?

**DOI:** 10.3389/fpsyg.2022.953402

**Published:** 2022-07-26

**Authors:** Isabel Krug, Matthew Fuller-Tyszkiewicz, Elizabeth K. Hughes, María Roncero

**Affiliations:** ^1^School of Psychological Sciences, The University of Melbourne, Parkville, VIC, Australia; ^2^School of Psychology, Deakin University, Geelong, VIC, Australia; ^3^Department of Paediatrics, University of Melbourne, Parkville, VIC, Australia; ^4^Murdoch Children's Research Institute, Parkville, VIC, Australia; ^5^Departamento de Personalidad, Evaluación y Tratamientos Psicológicos, Universitat de València, Valencia, Spain

**Keywords:** OSFED, UFED, Orthorexia, Bigorexia, Pregorexia, food addiction, Night Eating Syndrome

More than 50% of eating disorder (ED) patients are diagnosed as “Other Specified Feeding or Eating Disorder (OSFED)” or “Unspecified Feeding and Eating Disorders” (UFED). These two broad classifications within the Diagnostic and Statistical Manual of Mental Disorders (DMS-5) encompass heterogeneous ED symptom presentations. Purging Disorder (PD), Atypical Anorexia Nervosa (AAN), Night Eating Syndrome (NES), and Subthreshold Bulimia (BN)/Binge Eating Disorder (BED) are all designated presentations with the OSFED classification (American Psychiatric Association, 2013).

Other disordered eating problems, such as Orthorexia nervosa, Bigorexia, Drunkorexia, and Pregorexia (referred to as EXIAs hereafter), have recently received attention in the disordered eating/body image literature, but their recognition within an official classification system, such as the DSM-5 (American Psychiatric Association, 2013), is currently lacking. Thus, this Research Topic sought to gather evidence from researchers and clinicians working on projects focusing on the classification, risk assessment, and prevention/treatment of OSFED, UFED, and EXIAs.

We present a total of 5 entries for this topic, two of which focused on OSFED, two on Orthorexia, and one on food addiction. Though arguably tangential, a study by Pape et al. on food addiction, which is defined as a loss of control over eating, emotional eating, and food cravings, was included to provide an important asset to clarifying uncertainties around a range of disordered eating problems, not just the ones defined in the current Research Topic.

Kaur et al.'s systematic review found that NES patients had higher ED pathology and a higher prevalence of depressive symptoms than controls. The review also found an increased prevalence of emotional eating, body-related concerns, and atypical eating episodes in NES when compared to BED. The review concluded that more research into NES as a distinct condition is required, and that it is critical to develop appropriate diagnostic criteria and treatment options for NES.

Withnell et al. investigated differences in ED severity and general psychopathology in threshold ED and OSFED patients at intake and discharge to an ED unit. The results demonstrated that the OSFED and threshold ED groups had similar global ED symptoms, as well as shape and weight concerns. There were no differences in level of changes in self-esteem, depression ratings, or symptom change from entry to discharge across diagnostic groups. As a result, these data call into question the notion that OSFED is less severe and resistant to therapy than threshold EDs. However, it should be noted that the majority of the OSFED individuals (86%) were from an “other” OSFED category (e.g., food restriction and shape/weight concerns) rather than the distinct OSFED entities (e.g., AAN, PD, etc.) currently outlined in the DSM-5.

Two studies in the current Research Topic investigated Orthorexia. The first study (Mitrofanova et al.) explored the experience of individuals who followed an Orthorexia-like diet using Behavioral Reasoning Theory. The factors identified as contributing to the development of Orthorexia were social, rules/control, and ethical considerations. Participants also commented on how Orthorexia behaviors impacted on their social life and their desire for control. Upcoming diagnostic criteria for Orthorexia should therefore take into consideration the importance of a variety of reasons for a restricted diet.

The second Orthorexia study (Roncero et al.), examined personality in relation to two Orthorexia dimensions-pathological Orthorexia (OrNe) and adaptive healthy Orthorexia (HeOr) in participants from the general community. The study found that OrNe was linked to a disordered personality constellation defined by trouble controlling emotions and negative affect, as well as eccentricity, feeling exceptional, and having beliefs that differed from the norm. HeOr, on the other hand, was associated with a high level of responsibility, self-control, the ability to focus, and psychoticism. Future research should investigate how these distinct sets of personality constellations emerge for OrNe and HeOr by considering a longitudinal approach.

Finally, Pape et al. investigated the prevalence of food addiction, general psychopathology, and relationships between weight and addiction-related variables in people attending a weight loss programme who were overweight or obese but did not have BED or BN. The study found that food addiction was prevalent in 15% of participants, with females having a larger frequency (albeit not statistically significant) than males. Food addiction was linked to a higher BMI at baseline, low self-esteem, impulsive and emotional eating, weight bias internalization, and food-related inhibitory control deficiencies. Furthermore, associations between food addiction and the severity of depressive symptoms, internet use disorder, and psychological discomfort were discovered. The study concluded that, even after adjusting for BED and BN, a relevant subgroup of individuals with overweight or obesity experiences food addiction.

Overall, the findings of this collection of articles suggest that OSFEDs and EXIAs including NES, Orthorexia and food addiction are common, can be as severe and resistant to change after treatment as threshold EDs and are associated with other forms of psychopathology. To date, the characterization of these disordered eating problems has been insufficient to warrant separate classifications in the DSM-5. It is hoped that this Research Topic serves as a call to action, with further research resulting in improved classification, aetiological knowledge, and clinical care of patients with OSFED, UFED, and other EXIAs. In turn, we may see this translate into stand-alone diagnoses in future versions of the DSM.

## Author Contributions

All authors listed have made a substantial, direct, and intellectual contribution to the work and approved it for publication.

## Conflict of Interest

The authors declare that the research was conducted in the absence of any commercial or financial relationships that could be construed as a potential conflict of interest.

## Publisher's Note

All claims expressed in this article are solely those of the authors and do not necessarily represent those of their affiliated organizations, or those of the publisher, the editors and the reviewers. Any product that may be evaluated in this article, or claim that may be made by its manufacturer, is not guaranteed or endorsed by the publisher.
